# The effect of Greek mountain tea extract and wheat germ extract on peripheral blood flow and eicosanoid metabolism in mammals

**DOI:** 10.1515/med-2025-1192

**Published:** 2025-05-13

**Authors:** Licia Pantano, Kento Hiroki, Ashiyu Ono, Yuta Osada, Yasuyuki Fujii, Luigi Maiolino, Ursula M. Jacob, Gaetano Cammilleri, Berit Hippe, Gianluigi M. Lo Dico, Vincenzo Ferrantelli, Naomi Osakabe, Vittorio Calabrese

**Affiliations:** Food Department, Istituto Zooprofilattico Sperimentale della Sicilia, Palermo, 90129, Italy; Department of Bioscience and Engineering, Functional Control Systems, Graduate School of Engineering and Science, Shibaura Institute of Technology, 307 Fukasaku, Minumaku, Saitama, 337-8570, Japan; SIT Research Laboratories, Shibaura Institute of Technology, Saitama, Japan; Department of Medical, Surgical Advanced Technologies “G. F. Ingrassia”, University of Catania, Catania, Italy; Institute of Regeneration and Prevention, Healthcare AG, Zurich, Switzerland; Department of Nutritional Sciences, University of Vienna, Vienna, 1090, Austria; Department of Biomedical and Biotechnological Sciences, University of Catania, Catania, Italy

**Keywords:** Greek mountain tea, wheat germ, polyphenol, blood flow, eicosanoid

## Abstract

**Introduction:**

Polyphenols are a group of compounds identified as secondary metabolites of plants, with 8,000 types identified to date. Previous research findings have indicated the potential anti-inflammatory properties of polyphenols, with studies suggesting a reduction in disease risk and therapeutic benefits observed in various diseases, including diabesity, neurodegeneration, cancer, and cardiovascular disease.

**Objective:**

The objective of this study was to comprehensively analyze the polyphenol composition of extracts of Greek mountain tea (GMT) and wheat germ (WG) and investigate their effects on microcirculation and eicosanoid metabolism.

**Materials and methods:**

The polyphenol and spermidine composition of GMT and WGE was analyzed using LC–HRMS. Hemodynamic impact of GMT or WG on rat cremasteric arteriole blood flow was measured after compound administration using a laser Doppler blood flow meter. Lipidomic analysis in urine after co-administration of GMT and WGE was measured by LC–HRMS mass spectrometry.

**Results:**

This study shows that GMT contains large amount of polyphenols, expecially ferulic acid and petunidin. In contrast, in the WG extract we found minimal polyphenol content. Subsequent to the administration of GMT to rats, a significant increase in rat cremasteric arteriole blood flow was observed, while WG extract exhibited minimal change. Following a single oral administration of GMT or WG to mice, 24 h urine was analyzed for eicosanoids. A significant decrease in pro-inflammatory eicosanoids and a substantial increase in anti-inflammatory eicosanoids were observed in the treatment group compared with the control group.

**Conclusions:**

Given the established role of polyphenol intake in enhancing vascular endothelial function and increasing peripheral blood flow, we suggest that the observed increase in blood flow is a consequence of polyphenols in GMT. In contrast, the enhancement of eicosanoid balance was more pronounced in the WG extract group compared to the GMT group, suggesting that this effect may be attributable to components other than polyphenols present in these fractions.

## 1 Introduction

Polyphenols are a group of compounds that have been identified as secondary metabolites of plants, with 8,000 types identified to date [1]. Previous research findings have indicated the potential anti-inflammatory properties of polyphenols, with studies suggesting a reduction in disease risk and therapeutic benefits observed in various diseases, including diabetes [2] and obesity [3], neurodegeneration [4], cancer [5], and cardiovascular disease (CVD) [6].

Greek mountain tea (GMT; *Sideritis scardica*) is known as an herb rich in various bioactive compounds. The plant, which is indigenous to the rocky slopes of the Greek mountains, has been found to be rich in phytochemicals such as essential oil [7], flavonoids [8], terpenes [9], and polyphenols [10]. These compounds exhibit anti-inflammatory [11], antioxidant [12], and antibacterial effects [11], among others, contributing to its health-promoting properties.

Wheat germ (WG) is a by-product of wheat milling and is rich in antioxidants, including carotenoids, tocopherols, flavonoids, and phenolic acids [13]. The potential health benefits of WG are numerous, including a reduced risk of CVD [14], anti-obesity [15], and antidiabetic effects [16]. These benefits are thought to be partially due to the polyphenols contained in WG, but related research is limited.

In the circulatory system, polyphenols have been shown to promote nitric acid (NO) production, relax blood vessels, and protect the cells that make up the blood vessels [17,18]. Additionally, their regulatory function in the synthesis of eicosanoids [19,20], a group of oxygenated fatty acids with the capacity to induce inflammation, is considered to be significant in the context of vascular protection [19,20]. On the other hand, the effects of polyphenol on the metabolism of these eicosanoids remain unclear.

The present study was conducted with the objective of confirming the effect of polyphenols on vascular function and the effect of eicosanoids on the output. The focus was on GMT and WG extracts, and the polyphenols contained within them were comprehensively detected. The effects of these compositions on vascular function were observed in experimental animals using the intravital observation system, and the eicosanoids in the urine of mice administered were comprehensively analyzed.

## 2 Materials and methods

### 2.1 Materials

GMT and WG were kindly provided by System Biologie AG, Wollerau (Switzerland). Folin-Ciocalteu reagent, gallic acid, Tween 80, and urethane were obtained from Sigma Aldrich Japan (Tokyo, Japan).

### 2.2 Animals

All animals were humanely raised according to the ARRIVE guidelines of this period. All surgery was performed under anesthesia, and all efforts were made to minimize suffering. Fourteen male Wistar rats aged 7–8 weeks (200–280 g body weight), were obtained from Saitama Experimental Animal Supply (Tokyo, Japan). Sixteen 10 weeks old male C57BL/6J mice were obtained from CLEA Japan, Inc. (Tokyo, Japan). During 2 weeks acclimation period, the animals were carefully handled to reduce anxiety behaviors. Mice were housed at room temperature (24–26°C) under a 12 h light/dark cycle (light cycle: 7:00–19:00, dark cycle: 19:00–7:00) with free access to water and food. The solid diet (MF) for laboratory animals was obtained from Oriental Yeast Co., Ltd (Tokyo, Japan). The protocol was approved by the Animal Experimentation Committee of Shibaura Institute of Technology (approval number: AEA23009).

### 2.3 Analysis of polyphenols and spermidine in GMT and WG by the method of LC–Orbitrap-MS

The polyphenol and spermidine composition of GMT and WGE was analyzed using LC–HRMS as shown below, and the total polyphenol content of each fraction was quantified by the Folin-Ciocalteu method using gallic acid as a standard substance [21].

#### 2.3.1 Chemical reagents

The standard solutions of catechin, caffeic acid, syringic acid, rutin, hesperidin, ferulic acid, myricetin, quercetin, apigenin, naringenin, kaempferol, epigallocatechin, petunidin, procyanidin A2, procyanidin B2, cyanidin, pelargodin, petunidin, delphinidin, malvidin, and spermidine (each with a purity exceeding 99.9%) were obtained from Sigma-Aldrich S.r.l. (Milan, Italy), while chlorogenic acid was sourced from HWI Analytik GmbH (Rülzheim, Germany). A 10 mg portion of each powdered standard was dissolved in 10 mL of methanol to prepare solutions with a concentration of 1,000 mg L⁻¹. Notably, apigenin and kaempferol were dissolved in an aqueous solution with a pH greater than 8 (Alfano et al. [22]). Ultrapure deionized water was produced using a Milli-Q^®^ Integral water purification system from Millipore (Bedford, MA, USA). All reagents were of HPLC grade; acetone, acetonitrile, and formic acid were supplied by Sigma Aldrich (Amsterdam, Netherlands), and hydrochloric acid by Carlo Erba (Milan, Italy).

#### 2.3.2 Sample pretreatment and extraction method for polyphenols

The extraction protocol for phenolic compounds followed the method described by Puigventos et al. (2014; https://doi.org/10.1007/s00216-014-8298-2). Briefly 0.1 g of the sample of extracts was added to a 10 mL solution of acetone, water, and hydrochloric acid in a 70:29:0.1 v/v/v ratio. The mixture underwent sonication for 30 min, followed by centrifugation at 3,500 rpm for 15 min. The resulting extract was stored at −4°C. Before analysis, the extract was filtered using 0.45 μm nylon filters.

#### 2.3.3 LC–HRMS analysis for polyphenols

Chromatographic separations for phenolic compounds detection were carried out using a Raptor C18 column (2.1 mm × 100 mm, 1.7 μm). The mobile phase comprised eluent A (H_2_O + formic acid 1%) and eluent B (acetonitrile + formic acid 1%), with a total run time of 14 min and a flow rate of 0.3 mL/min. The chromatographic run initiates with 95% of A and 5% of B. Subsequently, there is a decrease in A and an increase in B by 25% within 0.33 min. Over the next 1.63 min, there is a continuous increase in B up to 100%. This condition is maintained for approximately 9 min. Finally, there is an increase in A and a decrease in B until the initial conditions are restored at 11.63 min. The quantification was conducted employing a Q-Exactive Plus Hybrid Quadrupole-Orbitrap™ Mass Spectrometer (Thermo Fisher Scientific, Waltham, MA, USA) in both positive and negative polarity modes. Polyphenol concentrations were expressed as mg/kg. The method underwent validation for linearity, recovery, repeatability, and reproducibility within the laboratory, following ISO/IEC 17025:2018, the Commission Regulation 836/2011 and the decision 657/2002/EC of the European Union. Limits of detection and limits of quantification (LOQs) were determined using the 3*σ* and 10*σ* approach (Lo Dico 2018; https://doi.org/10.1016/j.foodchem.2017.11.052).

#### 2.3.4 Sample extraction and detection of spermidine in WG extract

Spermidine extraction and detection was carried out according to the protocols proposed by Atikur Rahman et al. (2017; https://doi.org/10.4236/ajps.2017.812205). Briefly, 500 µL of a methanol/10 mM aqueous ammonium acetate solution (50:50) was added to the extract and vortexed for 1 min at room temperature. The mixture was then sonicated for 20 min and centrifuged at 17,000 × *g* for 10 min at 4°C. A 350 µL aliquot of the supernatant was transferred to 2 mL tube and evaporated under a gentle stream of nitrogen gas at 30°C. The dried residue was reconstituted in 100 µL of 0.1% aqueous formic acid. All the samples were analyzed in a Q-Exactive Plus Hybrid Quadrupole-Orbitrap™ Mass Spectrometer (Thermo Fisher Scientific, Waltham, MA, USA) in both positive and negative polarity modes. Chromatographic separation was carried out on a Raptor C18 column (2.1 mm × 100 mm, 1.7 μm). The flow rate was 350 µL/min and column temperature was 25°C. Total run time per sample was 21 min. The method was validated according to ISO/IEC 17025:2018, the Commission Regulation 836/2011 and the decision 657/2002/EC of the European Union.

### 2.4 Hemodynamic impact of GMT or WG on flow mediated vasodilation rat cremasteric arteriole blood flow

The animals were divided into two treatment groups: vehicle (4 mL/kg in 3% Tween 80 solution, *n* = 9), GMT (100 mg/kg, *n* = 9) or WG (100 mg/kg, *n* = 9). All chemicals were dissolved in a 3% Tween 80 solution. Blood flow measurement was performed according to previous report [23] as shown in Figure 2a. Briefly, rats were anesthetized with urethane (1 g/kg s.c.), and a gastric tube was inserted into the stomach. The cremaster muscle was exteriorized, and the surface was perfused with phosphate-buffered saline. After a post-surgical equilibration period, cremaster arteriole blood flow baseline measurements were conducted for 10 min. Each treatment was orally administered to animals through the gastric tube. Blood flow in the cremaster artery was monitored for 60 min after compound administration using a laser Doppler blood flow meter (Periscan PIM-2, Perimed Co. Ltd, Stockholm, Sweden).

### 2.5 Lipidomic analysis of mice urine after co-administration of GMT and WGE

It has been suggested that brief periods of social isolation in metabolic cages can markedly alter sympathetic nervous system activity in mice. In our preceding research, we demonstrated that co-housing two mice in metabolic cages markedly reduced the stress response associated with single housing [24]. Consequently, in the present study, we employed this approach to examine the eicosanoid metabolism after a single oral gavage administration of 100 mg/kg GMT or/and 100 mg/kg WG (*n* = 8). Following a 48 h acclimation period, urine was collected for 24 h using a tube containing 20 µL of 2.5 mol/L HCl following oral administration of test chemicals. Oral administration was performed between 10:00 and 11:00.

#### 2.5.1 Sample extraction and chromatographic analysis

**Figure 1:**
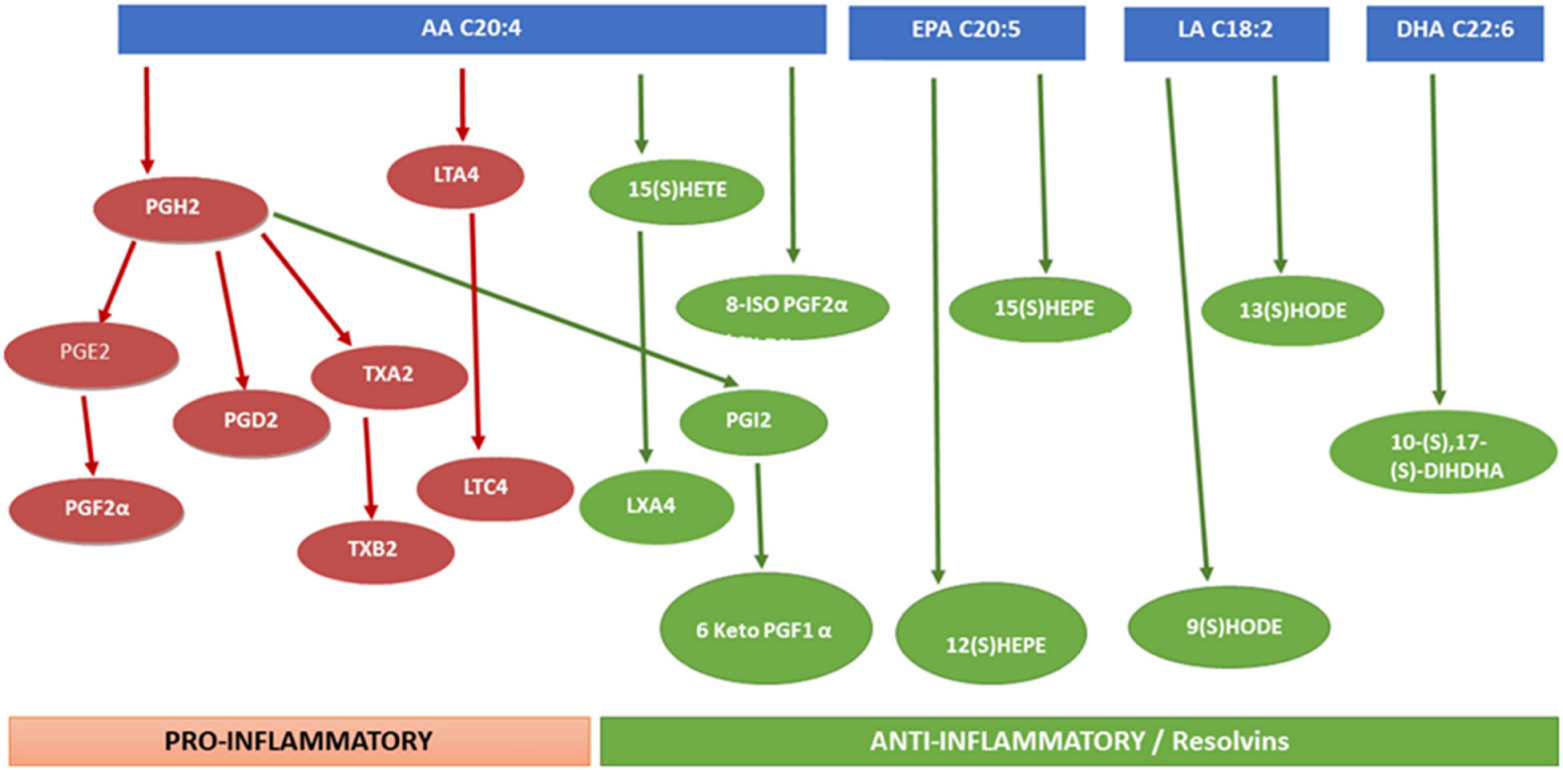
Scheme for mass spectrometry platforms that enable the quantification of diverse eicosanoid species in urine. AA, arachidonic acid; EPA, eicosapentaenoic acid; LA, linoleic acid; DHA, docosahexaenoic acid; LT, leukotriene; PG, prostaglandin; HEPE, hydroxy-eicosapentaenoic acid; HETE, hydroxy-eicosatetraenes; HODE, hydroxy-octadecadienoic acid; DIHDHA, dihydroxy-docosahexaenoic acids.

To comprehensively observe the effects of GMT and WG extracts on eicosanoid metabolism (Figure 1), the following analysis was carried out. The concentration of creatinine was measured using Laboassay creatinine (FUJIFILM Wako Pure Chemical Corporation).

Urinary eicosanoid contents were shown for each pair and their average values. The urine samples were produced in accordance with previously published techniques [25]. In brief, 50 µL of urine was vortexed with 50 µL of MeOH and 20 µL of IS working solution. The chromatographic separation was performed on a 25°C XBridge BEH C18 column (21 mm × 50 mm, 2.5 µm). The mobile phases were H_2_O + 0.1% formic acid (A) and acetonitrile + 0.1% formic acid (B), with a flow rate of 0.25 mL/min. The injection volume was 10 µL, and all analytes were eluted between 0.10 and 12 min with gradients ranging from 10 to 90% B.

#### 2.5.2 MS conditions and validation of the method

As a mass spectrometer, a Q ExactiveTM Plus Hybrid Quadrupole-OrbitrapTM (Thermo Fisher Scientific, California, USA) with a heated electrospray ionization source was employed (HESI-II). Wolfer et al. provided the source parameters. The Full MS scan/dd-MS2 mode was used to collect all data. The resolution of the Orbitrap was adjusted to 140,000 FWHM (scan range 200–800 *m*/*z*). For a maximum injection period of 100 ms, the automatic gain control was set to 1 × 10^6^ ions. The product ions were discovered by raising the normalized collision energy (NCE) until the precursor ions were completely fragmented. Each analyte was assigned a NCE value. The retention time (RT), accurate mass, and distinctive fragmentation were used to identify the analytes. All analyses were performed with no lock mass. Each day before the study, an external calibration for mass accuracy was done. Thermo Xcalibur TM version 4.0 software was used to record and expound on acquisition data. The method’s performance was evaluated for linearity, specificity, and trueness in compliance with Commission Decision 2002/657. The LOQ was computed as the smallest amount of standard required to generate an *S*/*N* > 5 while remaining within the calibration curve’s linear range (back-calculated residual 20%). The linearity test yielded good results for all analytes tested (*r* ^2^ > 0.997). Trueness by recovery yielded values ranging between 80 and 104%. This method allows simultaneous analysis of the following eicosanoids: prostaglandin F2α(PGF2α), prostaglandin E2(PGE2), prostaglandin D2 (PGD2), leukotriene C4 (LTC4), thromboxane B2 (TXB2), 11-dehydro thromboxane B2 (11-dehydro TXB2), 12-(*R*)-hydroxy-eicosatetraenes (12RHETE), 9-(*S*)-hydroxy-octadecadienoic acid (9SHODE), 15-(*S*)-hydroxy-eicosapentaenoic acid (15SHEPE), 12-(*S*)-hydroxy-eicosapentaenoic acid (12SHEPE), 13-(*S*)-hydroxy-octadecadienoic acid (13SHODE), 15-(*S*)-hydroxy-eicosatetraenes (15SHETE), 8-(*S*)-hydroxy-eicosatetraenes (8SHETE), 6-Keto prostaglandin F1α (6-Keto PGF1α), 10-(*S*),17-(*S*)-dihydroxy-docosahexaenoic acids (10S17SDIHDHA), and docosahexaenoyl ethanolamide.

### 2.6 Data analysis and statistical methods

The results of blood flow changes were expressed as the mean and standard deviation and statistical analysis was performed using two-way analysis of variance, and *post hoc* comparisons were made using Tukey’s test. All statistical analysis was conducted by using the software GraphPad Prism 10 (https://www.graphpad.com/features). The probability of *p* < 0.05 was considered significant. The urine excretion of each eicosanoid was expressed as a ratio with the urinary creatinine concentration.

## 3 Results

### 3.1 Concentration of polyphenols and spermidine in GMT and WG

The retention time (RT) and concentration of polyphenols and spermidine obtained in GMT and WGE samples are reported in Table 1. As reported, in GMT we found very high amount of ferulic acid, epigallocatechin and, in particular, petunidin. In WG we found at significantly high amount spermidin and apigenin. LC-MS analysis of WG and GMT extracts are shown, respectively, in Figure 3a and b.

**Table 1:** Polyphenol and spermidine composition of GMT and WG extracts

Compound	RT (min)	*m*/*z*	GMT (µg/g)	WG (µg/g)
Chlorogenic acid	2.80	355.10236	n.d.	8.965
Caffeic acid	3.11	181.04954	77.359	1.563
Rutin	4.10	611.16066	20.773	n.d.
Ferulic acid	3.50	195.06519	3927	n.d.
Quercetin	5.50	303.04993	16.135	n.d.
Apigenin	5.13	196.06519	n.d.	118.639
Epigallocatechin	6.30	307.08193	179.871	n.d.
Petunidin	7.18	317.06558	19159.426	n.d.
Spermidine	2.04	229.08592	n.d.	290200.37
Total polyphenols*			17.8	1.25

*Total polyphenols content was measured by the Folin method and was expressed in % (w/w) gallic acid equivalents. n.d. = not detected.

### 3.2 Hemodynamic impact of GMT or WG on rat cremasteric arteriole blood flow

**Figure 2:**
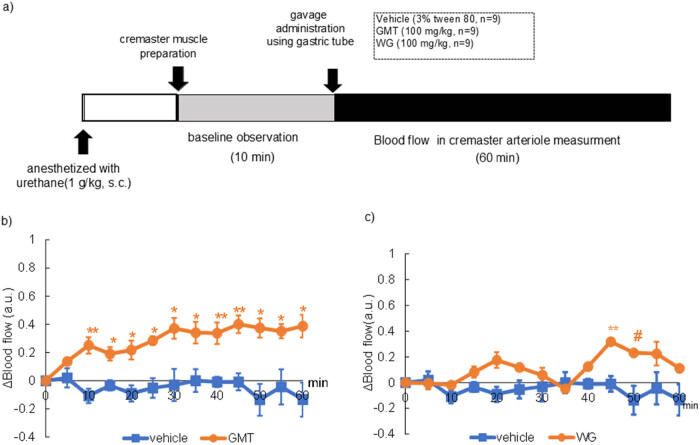
Change of blood flow in rat cremasteric arteriole GMT or WG. (a) Experimental procedure, (b) change in blood flow following a single gavage dose of vehicle (3% tween 80, *n* = 9) or 100 mg/kg GMT (*n* = 9), (c) change in blood flow following a single gavage dose of vehicle (3% tween 80, *n* = 9) or 100 mg/kg WG (*n* = 9). The values represent mean ± standard deviation. ^#^ *p* < 0.1, *p < 0.05, **p < 0.01, two-way ANOVA, followed by the *post hoc* Dunnett’s test, compared with vehicle.

The change of blood flow in rat cremaster arteriole after a gavage administration of 100 mg/kg GMT or WG extraction are shown in Figure 2b and c. Immediately after GMT was administered, blood flow began to increase, and showed a significant increase compared to the control group from 10 to 60 min (Figure 2b). In contrast, as reported in Figure 2c, a slight increase in blood flow was observed 45–50 min after administration of the WG extract.

### 3.3 Lipidomic analysis of mice urine after co-administration of GMT and WGE

The urinary excretion of the pro-inflammatory eicosanoids PGF2α, PGE2, TXB2, 11-dehydro TXB2, and 12RHETE was significantly decreased by administration of GMT or WG extract, and the effect was particularly strong with WG extract. Co-administration of these substances resulted in additive decreases. The urinary excretion of the anti-inflammatory eicosanoids 9SHODE, 13SHODE, 6-Keto PGF1α, and docosahexaenoyl ethanolamide was significantly increased by administration of GMT or WG extract, and the effect was particularly strong with WG extract. Co-administration of these substances resulted in additive increases. PGD2, LTC4 15SHEPE, 12SHEPE, 15SHETE, 8SHETE, 6-Keto PGF1 α, and 10S17SDIHDHA in the collected urine were below the detection limit (Figure 4).

## 4 Discussion

**Figure 3:**
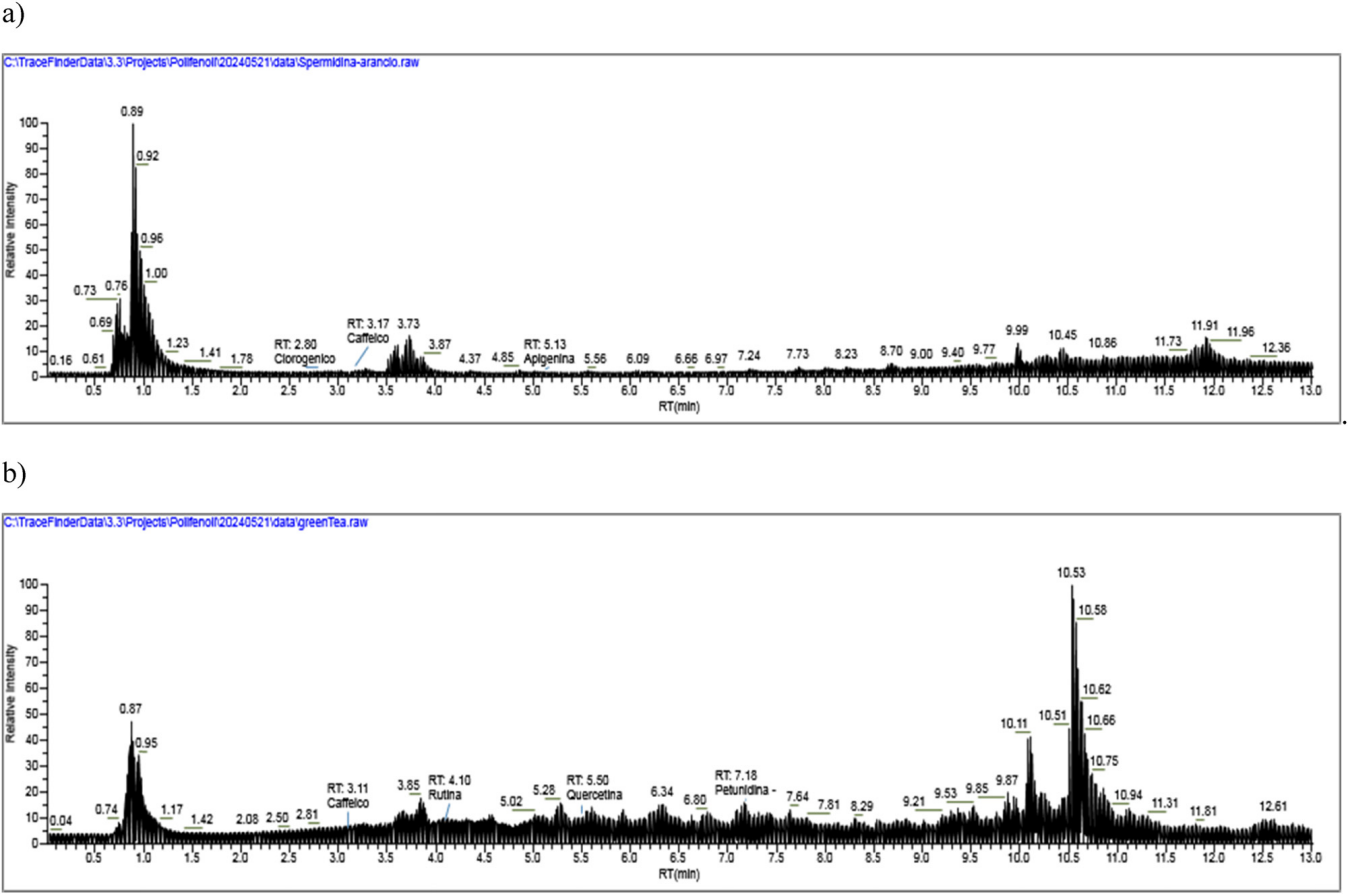
LC–HRMS chromatograms from WG (a) and GMT (b) extracts.

**Figure 4:**
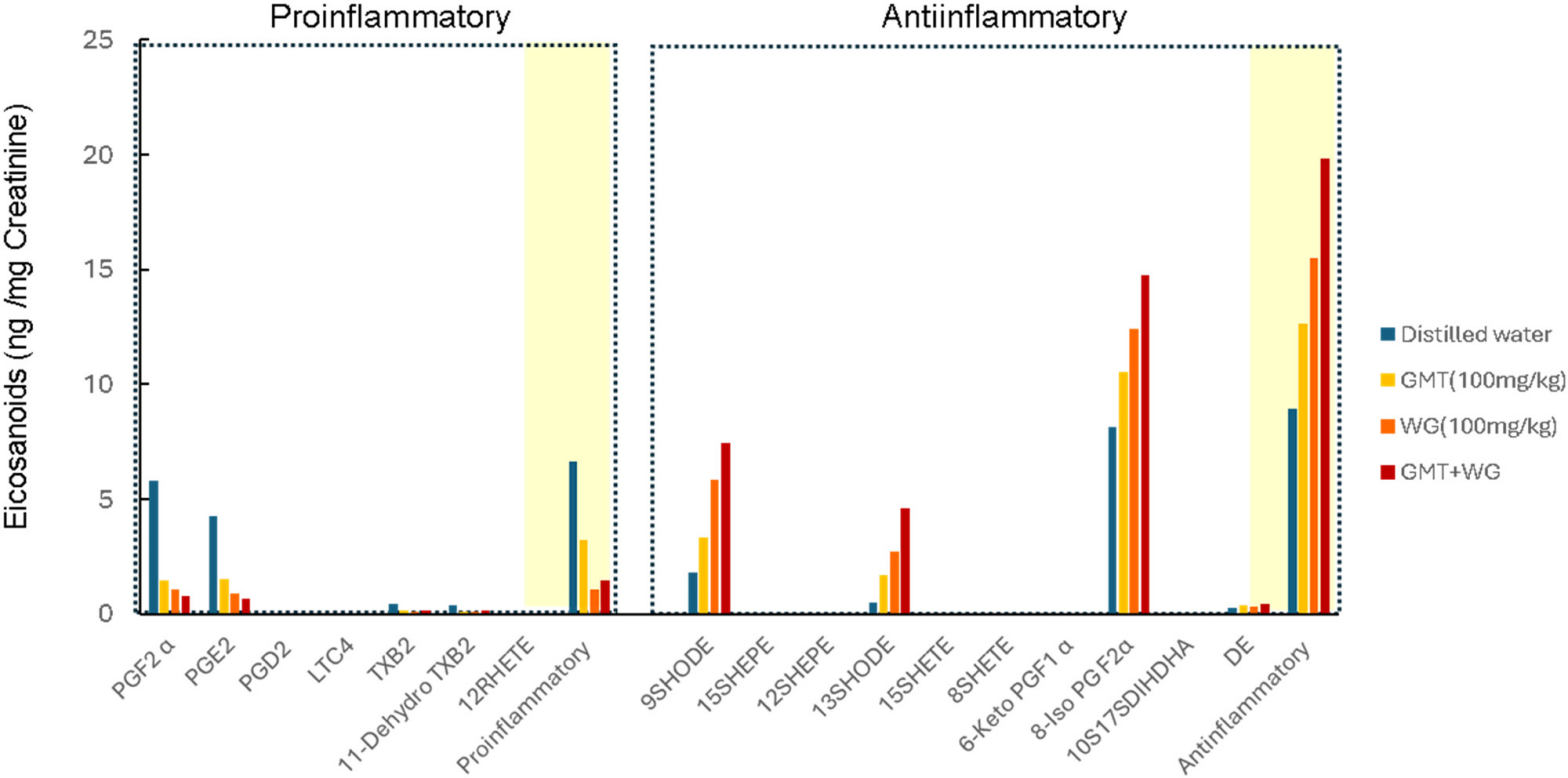
Change of total pro- or anti-inflammatory eicosanoids concentration in urine after a single gavage administration of GMT (100 mg/kg) or/and WG extract (100 mg/kg). Each value represents the average of ratio with the urinary creatinine concentration. LT, leukotriene; PG, prostaglandin; HEPE, hydroxy-eicosapentaenoic acid; HETE, hydroxy-eicosatetraenes; HODE, hydroxy-octadecadienoic acid; DIHDHA, dihydroxy-docosahexaenoic acids; DE, docosahexaenoyl ethanolamide.

A thorough investigation into the polyphenol composition of the GMT and WG extracts was conducted in this study, yielding notable findings. The analysis revealed the presence of substantial concentrations of ferulic acid, epigallocatechin and, in particular, petunidin in GMT, with the total polyphenol content being recorded at 17.8% (Table 1 and Figure 3a). In addition, following oral administration of GMT, a significant increase in blood flow was observed in the rat’s elevator muscle, which was sustained throughout the 60 min observation period (Figure 3). It is well established that flow-mediated vasodilation (FMD) increases 2 h after the ingestion of polyphenol-containing foods in numerous intervention studies, including those examining cocoa [26], grape [27], cranberry [28], and black tea [29]. These intervention studies have shown that NO metabolites, which are responsible for vasodilation, increase. It has been suggested that polyphenol intake immediately activates eNOS in the vascular endothelial cells, and the NO produced relaxes the vascular smooth muscle, increasing the blood flow, resulting in an increase in FMD values [30]. Decreased endothelial function reduces the flexibility of blood vessels and increases the blood pressure, which increases the risk of arteriosclerosis directly linked to CVD [31,32]. Previous epidemiological studies have demonstrated a strong negative correlation between the frequency of polyphenol intake and CVD risk [30]. Based on these findings, it is expected that the intake of GMT will improve the vascular function and reduce the risk of CVD. However, polyphenols are poorly absorbed from the digestive tract, so it is unlikely that they are distributed in the blood and have a direct effect on blood vessels [33]. We demonstrate here that GMT has high levels of antocyanidins. Accordingly, it is known that monomeric and dimeric procyanidins undergo some absorption within the gastrointestinal tract, while their larger oligomeric and polymeric counterparts are not bioavailable. However, higher molecular weight procyanidins engage with the colonic microbiota, fostering the production of bioavailable metabolites that undergo metabolic processes, culminating in the emergence of bioactive agents capable of modulating physiological processes. Consistent with this notion, flavonols are degraded by intestinal bacteria to produce a specific metabolite, phenyl-γ-valerolactone (gVLM). Therefore, many researchers believe that the various beneficial effects of flavonols may be mediated by these metabolites [34]. However, in mammals, it has been reported that FL reached two peaks in plasma concentration (*C*_max_) of 260 and 88 nmol/L gVLM at 1.8 and 5.3 h (*T*_max_) after ingestion [35]. In contrast, the WG extract employed in this study exhibited low levels of polyphenols compared with GMT (Table 1 and Figure 3b). Moreover, the augmentation in blood flow within the skeletal muscles of rats subsequent to the administration of WG extract was slight in comparison to GMT. These outcomes are presumably indicative of discrepancies in polyphenol content. Conversely, a comparison of urinary eicosanoid excretion after single administration of GMT and WG extracts to mice revealed some notable findings. All pro-inflammatory eicosanoids were decreased by administration of GMT or WG, but this effect was more pronounced with WG than with GMT. Conversely, all anti-inflammatory eicosanoids were significantly increased by administration of GMT or WG, but this effect was more pronounced with WG than with GMT. Furthermore, in the combination study, the balance between pro-inflammatory and anti-inflammatory eicosanoids was improved by the combination of GMT and WG (Figure 4, Table 1). These results suggest that the mechanism of action of WG extract on eicosanoid metabolism may be brought about by components other than the different polyphenols contained in WG.

GMT or/and WG extract showed to decrease the *in vivo* synthesis of PGE2 and TXA2, as evidenced by the reduced urinary excretion of their metabolites, PGF2α and TXB2 (Figure 4, Table 1). It is well established that PGE2, which is produced in significant quantities at pro-inflammatory sites, is a major mediator of inflammation and functions as a key protein in the generation of inflammatory pain [36,37]. Furthermore, TXA2 was recognized for its potent vasoconstricting action and platelet activation, which contributes to thrombus formation during periods of tissue damage or inflammation [38]. The capacity to inhibit the production of these pro-inflammatory eicosanoids offers significant potential for the regulation of a range of inflammatory conditions, including cardiovascular or neurodegenerative diseases. Furthermore, the urinary excretion of 9HODE and 13HODE was increased by GMT or/and WG extract. HODEs have heretofore been considered to be biomarkers reflecting increased oxidative stress in various diseases [39,40]. Conversely, it has been reported that in early atherosclerosis, 13-HODE, produced by macrophages, activates peroxisome proliferator-activated receptor γ and promotes the removal of lipids and lipid-laden cells from the arterial wall [41,42]. In addition, it has been hypothesized that both 9HODE and 13HODE may enhance the apoptosis sensitivity of vascular cells and suppress the formation of atherosclerotic plaques [41,43]. Consequently, these findings suggest that the alterations in eicosanoid balance induced by oral ingestion of GMT and WG may be vascular protective. Although, presently, our experiments did not clarify the components in GMT and WG extracts that regulate eicosanoid metabolism and, in addition, due to the analytical approach, we cannot exclude the possibility that water-soluble components in GMT and WG extracts might contribute also to the observed regulation of eicosanoid balance. We here observed that GMT and WG extracts tended to suppress the production of pro-inflammatory eicosanoids and promote the production of anti-inflammatory eicosanoids, yet the components responsible for this beneficial effect remain unknown. Further comprehensive analysis of the components contained in GMT and WG extracts would clarify better the mechanism of action associated with pro-health effect of our tested extracts. It is also important to conduct long-term animal studies to clarify the cardiovascular and neuroprotective benefits of GMT and WG extracts and to link them to intervention studies.

In conclusion, we found that GMT contains a wealth of polyphenols and enhances vascular endothelial function immediately after administration, and that GMT and WG extracts contain powerful eicosanoid balance-regulating components in addition to polyphenols. Further studies are needed to clarify the cardiovascular and neurodegenerative disease risk reduction effects of GMT and WG extracts.
